# Suppression durations for facial expressions under breaking continuous flash suppression: effects of faces’ low-level image properties

**DOI:** 10.1038/s41598-020-74369-2

**Published:** 2020-10-15

**Authors:** Abigail L. M. Webb, Paul B. Hibbard

**Affiliations:** Department of Psychology, University of Essex, Wivenhoe Park, Colchester, CO4 3SQ UK

**Keywords:** Human behaviour, Visual system

## Abstract

Perceptual biases for fearful facial expressions are observed across many studies. According to the low-level, visual-based account of these biases, fear expressions are advantaged in some way due to their image properties, such as low spatial frequency content. However, there is a degree of empirical disagreement regarding the range of spatial frequency information responsible for perceptual biases. Breaking continuous flash suppression (b. CFS) has explored these effects, showing similar biases for detecting fearful facial expressions. Recent findings from a b. CFS study highlight the role of high, rather than low spatial frequency content in determining faces’ visibility. The present study contributes to ongoing discussions regarding the efficacy of b. CFS, and shows that the visibility of facial expressions vary according to how they are normalised for physical contrast and spatially filtered. Findings show that physical contrast normalisation facilitates fear’s detectability under b. CFS more than when normalised for apparent contrast, and that this effect is most pronounced when faces are high frequency filtered. Moreover, normalising faces’ perceived contrast does not guarantee equality between expressions’ visibility under b. CFS. Findings have important implications for the use of contrast normalisation, particularly regarding the extent to which contrast normalisation facilitates fear bias effects.

## Introduction

According to the threat bias theory, the visual system has evolved specialised mechanisms for detecting and responding to stimuli containing threat-relevant information^1–3^. An example of this is the idea that there exist perceptual biases for fearful expressions, since they provide indirect signals of a potential threat shared by the observer and the expresser^2,4,5^. Evidence for these perceptual biases come from a range of experimental paradigms. Fear expressions are associated with enhanced orientation of visual spatial attention, which has been found with both manual detection times for, and rapid eye movements towards, fearful expressions^6–8^. Prior exposure to fearful expressions also improves search efficiency^9,10^, and can selectively enhance even very simple aspects of early visual processing such as orientation discrimination^11–13^. Evidence from neuroimaging studies implicates subcortical threat-processing mechanisms in responding to expressions of fear, consistent with the notion that a threat bias for fear expressions may operate via rapid, unconscious neural pathways^2,14–16^.

Low-level accounts of the fear bias propose that the biases in favour of fear expressions stem from their simple image characteristics, rather than the way in which their affective attributes and relevance are extracted and evaluated^8,17,18^. Converging evidence from psychophysical and physiological studies show that, in particular, perceptual biases for fearful expressions are driven by their low spatial frequency content. Low spatial frequency information conveys a coarse representation of the changes in light intensity occurring across an image. A low-pass filtered image contains information that is sufficient for interpreting global and indistinct image features, but offers little information regarding finer, more defined image qualities. The latter information is instead conveyed by higher spatial frequency information^8,11,13^. When low-pass filtered to contain only low spatial frequency information, fear expressions elicit faster saccadic eye movements^8^, improved performance and faster responses on subsequent attentional orientation tasks^12,19^. They also elicit larger and quicker responses from subcortical regions, including the amygdala, which is thought to underpin automated threat-avoidance responses^20–22^.

Breaking continuous flash suppression (b. CFS), a form of binocular rivalry, is an increasingly popular technique that has been used to explore the threat bias, and in particular how different ranges of spatial frequency contribute to the visibility of different facial expressions^23^. Under b. CFS, a dynamic masking stimulus is presented to one eye, and its function is to suppress conscious detection and appraisal of a target image (such a fearful face) synchronously presented to the other eye. Target stimuli therefore compete for conscious perception against suppressing masks. Under b. CFS, detection times to indicate the presence of a fearful expression are shorter than those for other facial expressions^17,18,24,25^. Using b. CFS, Stein et al.^25^ showed that, when highpass filtered to contain only high spatial frequencies, fearful faces broke suppression more quickly than neutral expressions. In contrast, no differences between expressions were found when faces were lowpass filtered to contain only low spatial frequencies. This finding directly contrasts with other psychophysical and physiological findings, which have identified that the threat bias is driven by low spatial frequencies.

One possible explanation for the discordance of these findings is the role of b. CFS-related effects on stimulus visibility. Cumulative evidence shows that suppression strength varies according to multiple factors under b. CFS, including the spatial and temporal properties of target and mask stimuli. For example, findings show that the suppression strength of b. CFS masks are strongest temporal frequencies below that of 10 Hz that is typically used^26,27^, when target stimuli and masks are matched for temporal frequency^28^, and also according the spatial noise used to create masks^29^.

Yang and Blake^24^ show that the spectral composition of both target and mask are also predictors of suppression strength. Masks consisted of collages of randomly positioned rectangles, and were assigned a 1/f amplitude spectrum, such that contrast amplitude in the mask decreased with increasing spatial frequency. The targets were either narrow-band Gabor stimuli, or faces with neutral expressions, filtered to contain only frequencies either below 0.75 cpd (low frequency), or above 6 cpd (high frequency). For the Gabor stimuli, they found that the degree of masking peaked at 1 cpd. For the face stimuli, masking was greater for low spatial frequency versions of faces. These results reflect the combination of the 1/f amplitude spectrum of the mask used, and the contrast sensitivity function of human vision. The degree of masking for both faces and simple Gabor stimuli thus appears to be determined by the effective contrast of the mask and target. Better suppression for low frequency stimuli evidenced by Yang and Blake^24^ offers an account of the detection advantage for high frequency fear expressions observed by Stein et al.^25^. Stein et al.^25^ employed fearful and neutral face images, and these were presented in original, broadband format, or filtered to contain only low (< 2 cpd) or only high (> 6 cpd) spatial frequencies. There were three conditions for masks: (1) an original mask composed of a collage of randomly positioned circles, with a 1/f amplitude spectrum (similar to that used by Yang and Blake^24^), (2) a hybrid mask composed of the sum of low- and high-pass filtered version of the original mask, matched across the two frequency bands for RMS contrast, and (3) only a highpass filtered version of the mask was used. Suppression strength was greater for low frequency faces when original masks were used. This is consistent with the greater amplitude of masking energy within the low frequency range. In all conditions, the degree of suppression was greater for fearful than neutral faces, but only for targets filtered to contain just low spatial frequencies. Great caution is required when interpreting these effects, however. The visibility of, and interaction between, the mask and target may be driven by spatial frequency channels outside the bandwidth of the target stimuli themselves^30–32^. In addition, both Yang and Blake^24^ and Lunghi and Alais^33^ show that suppression effects are not equal across spatial frequencies, but disproportionately stronger for low compared to high spatial frequencies. It is notable in particular that substantial suppression was found by Stein et al.^25^ when the mask was high-pass filtered and the target lowpass filtered. Moreover, the role of image contrast is relevant here, too. Contrast refers to the size of the difference between the brightest and darkest points within an image, and as such, is a determinant of an image’s salience. Contrast normalisation is a frequently used procedure for reducing physical differences between stimuli, therefore reducing inadvertent differences between images’ salience. Although faces used by Stein et al.^25^ were normalised for contrast, they were not considered in terms of their apparent, perceived contrast. Apparent contrast refers not to the physical contrast of an image, but its subjective appearance, and is an important consideration for broadband stimuli^34,35^. This is complicated by the fact that fearful and neutral stimuli differ in their overall contrast^36,37^ and Fourier amplitude spectra, with fearful faces tending to contain relatively more contrast energy at high frequencies in comparison with neutral expressions^37^. Moreover, broadband fear expressions less physical root mean squared (RMS) contrast compared to other expressions, particularly as their spatial frequency content is increased^37^. Importantly, however, the same effects were not the same when Michelson contrast was the metric used, highlighting the importance of consideration that is given to the metric for contrast^37^. It is important to control for and understand these variations, particularly given that several low-level explanations of the threat bias implicate the role of image properties known to influence salience, including contrast and spatial frequency. As such, bias in the perception of fearful faces relative to other expressions should be eliminated when stimuli are normalised for their apparent, perceived contrast, rather than their physical contrast alone.

It is also important to compare biases for facial displays under b. CFS to those from other studies using different paradigms, particularly those which suggest that the threat-bias is mediated by subcortical mechanisms, and driven by low spatial frequency information^2,6–8,11–16,20–22^. Together, these results would mean that the threat bias in effect pervades all levels of the visual hierarchy, and is driven by different frequency bands at different stages. The various tasks used in behavioural studies then tap into this bias at different stages. Stein et al.^25^ argue that b. CFS is a measure of the bias at higher levels of processing, within cortical areas, while other tasks measure the effects in lower subcortical areas, driven by low spatial frequencies. Finally, there is also a growing body of findings to show that sensitivity under b. CFS varies according to temporal frequency mask properties^26,27,33^, is highly sensitive to stimulus parameters^24^, and produces results with large individual differences^38^. Moreover, to our knowledge this is the only study to show a high spatial frequency-dependent fear detection bias, and has not yet been replicated by other researchers.

The purpose of the present experiment is to replicate and extend the study design employed by Stein et al.^25^, in order to understand the spatial information that underpins the threat bias in b. CFS. This extension contributes to our understanding of how low-level image properties influence perceptual biases for face expressions, how these effects manifest under b. CFS conditions, and what this means for the value of this technique as a measure of conscious perceptual biases. We used the same experimental parameters as those employed by Stein et al.^25^, but extended this to include (1) a broader range of facial expressions, including happy, angry and disgust stimuli (2) a mid-range spatial frequency condition as an intermediate between the low and high frequency conditions, to better understand the frequency tuning of suppression and (3) faces matched for both physical *and* perceived contrast.

## Methods

### Participants

Twenty-nine participants took part in the first study (broadband stimuli). Seventeen additional participants took part in the remaining conditions (low-, mid- and high-frequency stimuli). All participated in the experiment as part of a credited research module assessment. All participants had normal to corrected vision. The University of Essex Ethics Committee approved the study on the grounds that the study design was in accordance with university ethical guidelines and regulations. All participants were told that the study was concerned with face perception, and all gave written, informed consent.

### Stimuli and apparatus

Stimuli were presented using a VIEWPIXX 3D monitor, viewed from a distance of 80 cm. A chin rest was used to maintain viewing distance and eye-level. The monitor screen was 52 cm wide by 29 cm tall. The screen resolution was 1920 × 1080 pixels, with a refresh rate of 120 Hz, and an average luminance of 50 cdm^2^. All stimuli, including masks, were generated and presented using MATLAB and Psychophysics Toolbox extensions, and were delivered via NVIDIA 3D vision liquid–crystal shutter goggles^39–41^. Note, that the use of shutter goggles in the present study differs from the mirror stereoscope used by Stein et al.^25^.

#### Face stimuli

Stimuli were grayscale front-view face photographs of 16 actors (eight women, eight men) extracted from the Karolinska Direct Emotional Faces set^42^. Faces were cropped to include only internal features. Each actor portrayed a neutral, angry, fearful, happy or disgusted expression. The width of each face image was 4.5°. In MATLAB, a second-order Butterworth filter was used to create spatially filtered versions of the original, broadband images. The cut-off frequencies were *f* < 1_cpd_ for low spatial frequency (LSF) faces, 1 < *f* < 6_cpd_ for midrange spatial frequency (MSF) faces, and *f* > 6_cpd_ for high spatial frequency (HSF) faces. The frequency content of stimuli therefore varied between 4.5 and 27 cycles per face-width, and bandpass cut-offs were comparable to those used by Stein et al.^25^ and Vlamings et al.^20^. Faces were presented in two contrast formats: one in which they were normalised for root mean squared (RMS) contrast, and one in which they were psychophysically matched for perceived contrast. The latter contrast condition meant that faces were presented to observers with an associated Michelson contrast required for them to *appear* the same contrast. To create these faces, we utilised data from a separate study where a sample of participants (not associated with the present study) adjusted the physical contrast of the same grayscale 16 KDEF facial stimuli until they were perceptually the same^37^. This provided the 16 KDEF faces used in the present study with an assigned Michelson contrast value, corresponding to the degree of physical contrast necessary for them to perceptually match a reference face composed of 10% Michelson contrast. Therefore, faces matched for apparent contrast in the present study contained the degree of physical contrast required in order to subjectively appear as though they were composed of 10% Michelson contrast.

Faces were presented in a normal, upright format or as control versions. To create these control stimuli, images were spatially inverted (rotation by 180°) and their luminance polarity was reversed. Combining inversion and luminance polarity reversal reduces emotional recognition beyond that associated with inversion alone^17^. Doing so is therefore a useful tool for disrupting configural, face-specific processing, while preserving low-level image properties including contrast and spatial frequency content^17,43^.

#### Mask stimuli

**Figure 1:**
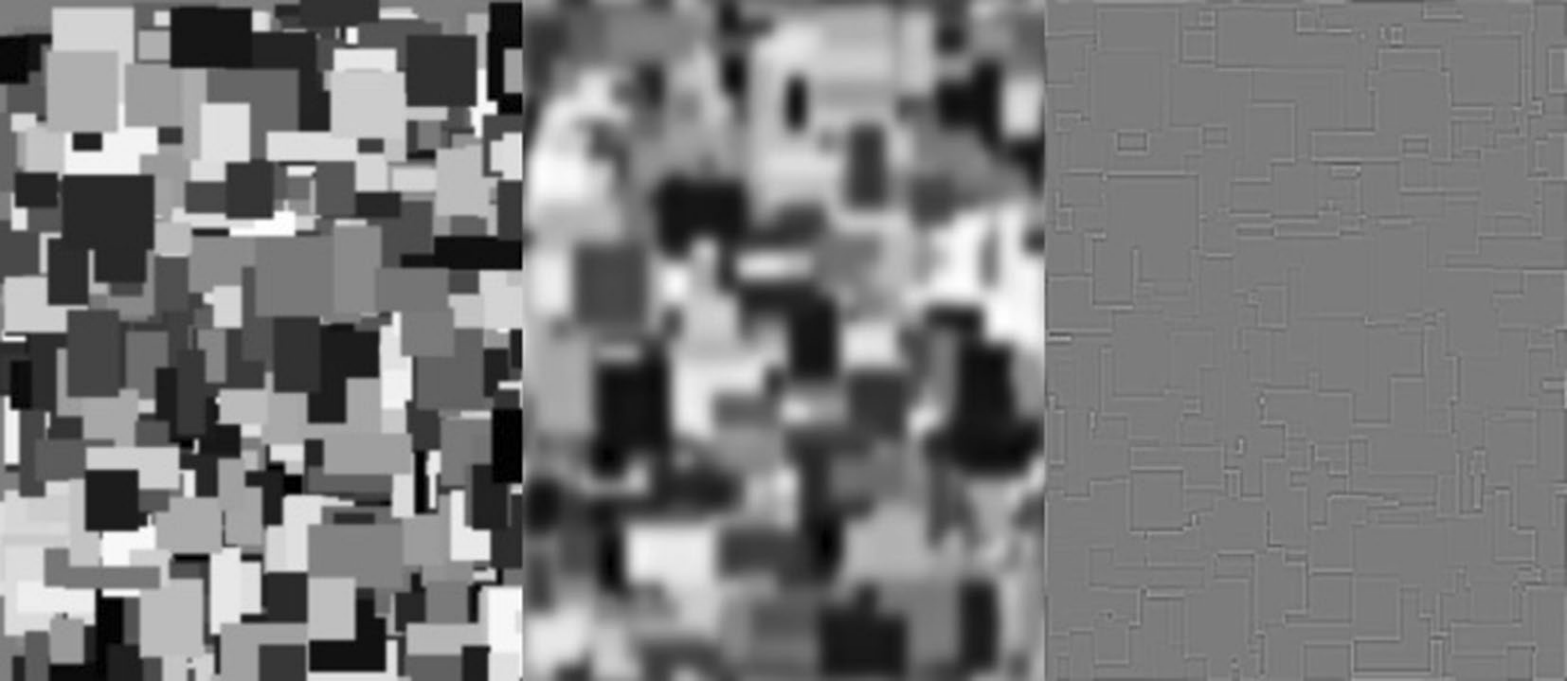
b. CFS masks, each composed of a rectangle collage, with a 1/f amplitude spectrum. Exemplars of masks at each frequency condition (left to right): intact broadband, lowpass filtered, and highpass filtered.

The same second-order Butterworth filters used to create the face stimuli were also used to create the b. CFS masks. Masks were composed of randomly positioned rectangles, with minimum and maximum widths and heights of 5.2 and 25 arcmin (respectively), with new samples presented at a rate of 10 Hz. On each trial, the spatial frequency content of masks and facial stimuli were matched. An example is shown in Fig. 1.

### Procedure

Participants were tested individually in a quiet room. Nvidia 3D goggles were used to present separate images to the two eyes. Note that Stein et al.^25^ used a mirror-stereoscope. Masks were present at full contrast for the duration of all trials, and face stimuli were presented individually at 1 of 4 quadrant locations. Faces reached full Michelson contrast 1 s after stimulus onset. Using a four alternative-forced-choice-task (4AFC), participants were instructed to indicate in which of the four quadrants each face was located, as quickly as possible. Manual responses were recorded using the RESPONSEPixx response box. Next trial onset was triggered by the observer’s response, but if responses were not made by 7 s post-trial onset, the next trial began. Overall, the study was separated into two parts. The first part of the study presented 29 observers with broadband facial stimuli: observers completed 320 trials (16 actors × 5 expressions × 2 contrast conditions × 2 orientations). Trials were randomised, and separated into eight blocks. The second part of the study presented 17 observers with low-, mid-, and high-frequency facial stimuli: observers completed 320 trials (16 actors × 5 expressions × 2 contrast conditions × 2 orientations). Trials were randomised, and separated into eight blocks. For both parts of the study, stimulus and procedural details were the same across each of the four studies, except for the spatial frequency content of faces.

## Results

Response times (RTs) reflect the point at which face stimulus broke suppression from b. CFS masks. Response times for each spatial frequency study (broadband, LSF, MSF, and HSF) were analysed separately. Here, each analysis included a 5 (Expression) × 2 (Contrast condition) × 2 (Orientation) repeated measures analysis of variance (ANOVA), and were followed by eight Šidák-corrected paired comparisons where appropriate (α = 0.0063, according to eight comparisons). Šidák corrections were selected over Bonferroni corrections for Comparisons explored differences in response times between fear and each counterpart expression, and were performed *separately* for each contrast condition.

### Response times for broadband faces

A repeated measures ANOVA showed significant effects of facial expression and face orientation on RTs, but no effect of contrast metric [*F*(4, 112) = 22.59, *p* < 0.001, η_p_^2^ 0.44; *F*(1, 28) = 22.20, *p* < 0.001, η_p_^2^ 0.44; *F*(1, 28) = 2.10, *p* 0.15, η_p_^2^ 0.07, respectively]. There was a significant contrast-orientation interaction [*F*(1, 28) = 4.74, *p* = 0.03, η_p_^2^ 0.14]. No significant contrast-expression, expression-orientation, or 3-way interactions were observed [*F*(4, 112) = 0.80, *p* 0.52, η_p_^2^ 0.02; *F*(4, 112) = 2.18, *p* 0.07, η_p_^2^ 0.02; *F*(4, 112) = 1.62, *p* 0.17, η_p_^2^ 0.05].

**Figure 2:**
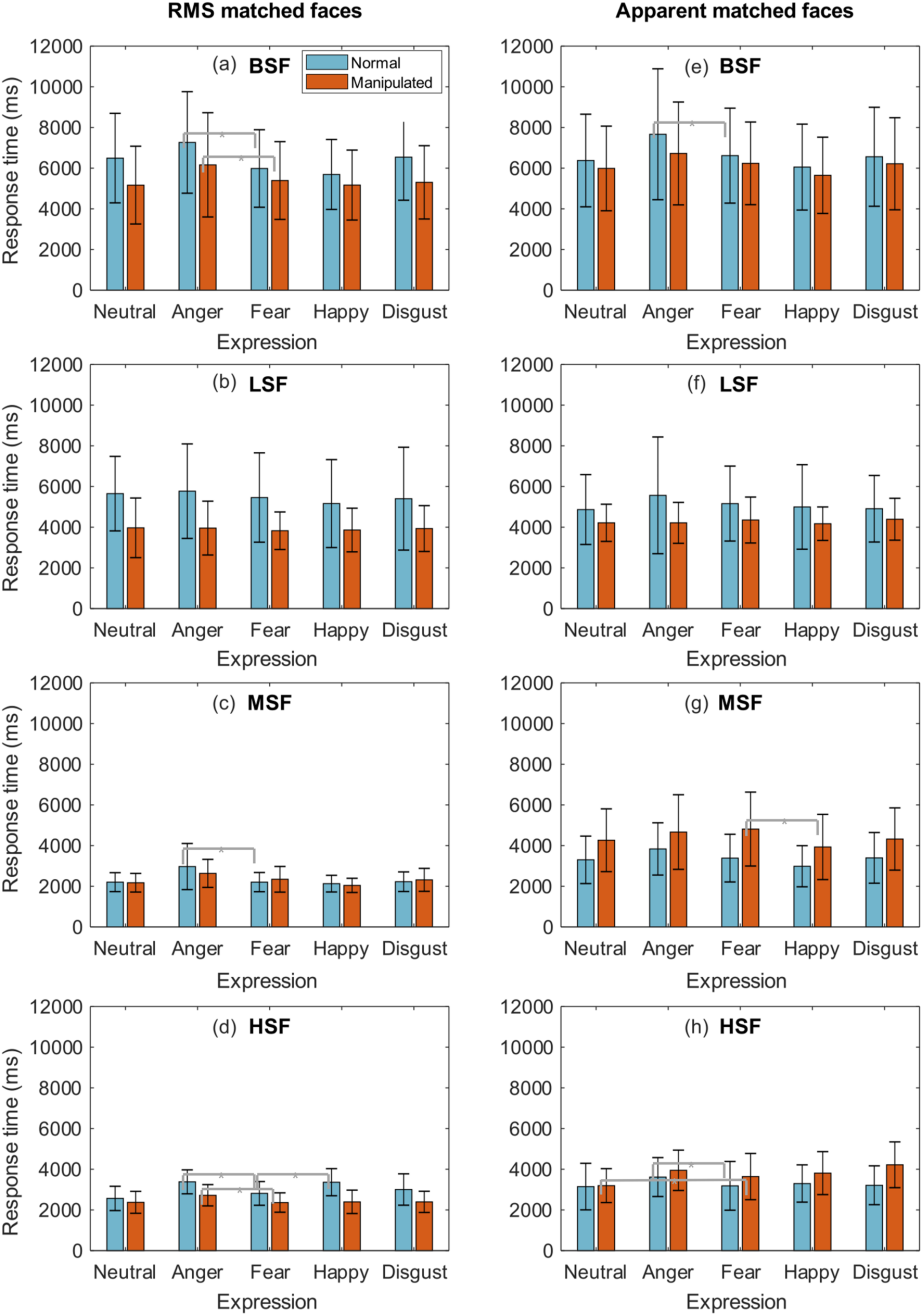
Response times (milliseconds) presented in multiple panels. Left column displays response times for faces normalised for RMS contrast, at each spatial frequency condition: (**a**) intact broadband, (**b**) lowpass filtered, (**c**) mid-range frequency filtered, (**d**) highpass filtered. Response times for the same frequency conditions are shown in the right column for faces that were normalised for apparent, perceived contrast. All error bars represent standard deviations. Asterisks denote statistically significant differences (α = 0.0063).

Expression-related differences in RTs were explored separately for faces normalised for RMS and apparent contrast. Eight Šidák-corrected tests (α = 0.0063) compared RTs for fear to each other expression, both when they were presented at normal, upright orientation, and when in control format. When normalised for RMS contrast, normal (non-control) fearful expressions were detected faster than angry faces (*p* < 0.001); an effect that remained true for control faces (*p* 0.005). When normalised for apparent contrast, RTs for normal fearful expressions were detected faster compared to angry expressions (*p* 0.003), but this effect was not found for control versions of faces. No other significant differences were observed. All comparisons are summarised in Table 1, and illustrated in Fig. 2a,e.

**Table 1:** Visibility differences between broadband expressions normalised for contrast.

	*t*	*df*	*CI*	*p*
**Expression comparisons (RMS)**				
Fear–neutral	− 2.45	28	− 933.12, − 84.68	0.02
Fear–anger	− 5.59	28	− 1748.20, − 811.27	< 0.001
Fear–happy	1.70	28	− 59.44, 646.64	0.10
Fear–disgust	− 2.27	28	− 1053, − 56.20	0.03
**Control comparisons (RMS)**				
Fear–neutral	0.91	28	− 282.66, 736.11	0.37
Fear–anger	− 2.98	28	− 1298.65, − 240.71	0.005
Fear–happy	0.92	28	− 272.62, 719.46	0.36
Fear–disgust	0.47	28	− 296.50, 476.39	0.63
**Expression comparisons (apparent)**				
Fear–neutral	0.97	28	− 267.30, 748.63	0.34
Fear–anger	− 3.19	28	− 1724.45, − 377.41	0.003
Fear–happy	1.80	28	− 76.80, 1204.39	0.08
Fear–disgust	0.18	28	− 572.64, 684.56	0.85
**Control comparisons (apparent)**				
Fear–neutral	1.07	28	− 227.53, 728.68	0.29
Fear–anger	− 1.69	28	− 1073.34, 100.64	0.10
Fear–happy	2.54	28	115.63, 1063.52	0.01
Fear–disgust	0.09	28	− 414.32, 454.26	0.92

Pairwise comparisons conducted separately for faces normalised for RMS contrast and those normalised for apparent, perceived contrast. In each contrast condition, eight comparisons compared response times between upright fear and counterpart expressions (4) and again for control versions of faces (4). All comparisons were Šidák-corrected according to eight comparisons: α = 0.0063.

In summary, for broadband stimuli, fearful faces were only detected more quickly than angry faces. This was true for both normal and control stimuli when stimuli were matched for RMS contrast, and for normal faces only when matched for apparent contrast.

### Response times for LSF faces

A repeated measures ANOVA showed significant effects of facial expression and face orientation on RTs, but no effect of contrast metric [*F*(4, 64) = 3.85, *p* 0.007, η_p_^2^ 0.19; *F*(1, 16) = 16.71, *p* 0.001, η_p_^2^ 0.51; *F*(1, 16) = 0.004, *p* 0.94, η_p_^2^ 0.00, respectively]. Again, a significant contrast-orientation interaction was observed. No significant contrast-expression, expression-orientation, or 3-way interactions were observed [*F*(4, 64) = 1.11, *p* 0.35, η_p_^2^ 0.06; *F*(4, 64) = 1.68, *p* 0.16, η_p_^2^ 0.09; *F*(4, 64) = 0.67, *p* 0.61, η_p_^2^ 0.04, respectively].

Eight Šidák-corrected tests (α = 0.0063) compared RTs for fear to each other expression, both for upright and control faces. Overall, RTs did not significantly differ between fear and any other expression, regardless of how they were normalised for contrast. No further analyses were conducted. All comparisons are summarised in Table 2, and illustrated in Fig. 2b,f.

**Table 2:** Visibility differences between low frequency expressions normalised for contrast.

	*t*	*df*	*CI*	*p*
**Expression comparisons (RMS)**				
Fear–neutral	− 0.70	16	− 689.48, 334.13	0.48
Fear–anger	− 1.41	16	− 967.62, 194.58	0.17
Fear–happy	2.09	16	87.06, 557.78	0.01
Fear–disgust	0.05	16	− 459.62, 484.37	0.95
**Control comparisons (RMS)**				
Fear–neutral	− 0.98	16	− 496.98, 181.78	0.34
Fear–anger	− 1.00	16	− 391.200, 139.23	0.32
Fear–happy	− 0.14	16	− 389.13, 338.64	0.88
Fear–disgust	− 1.07	16	− 256.26, 83.71	0.29
**Expression comparisons (apparent)**				
Fear–neutral	1.24	16	− 197.22, 753.59	0.23
Fear–anger	− 1.01	16	− 1338.74, 472.81	0.32
Fear–happy	0.87	16	− 274.31, 657.39	0.39
Fear–disgust	1.58	16	− 90.01, 621.63	0.13
**Control comparisons (apparent)**				
Fear–neutral	1.72	16	− 41.44, 407.38	0.10
Fear–anger	1.49	16	− 75.68, 436.22	0.15
Fear–happy	1.19	16	− 139.61, 499.90	0.25
Fear–disgust	− 0.17	16	− 313.56, 265.28	0.86

Pairwise comparisons conducted separately for faces normalised for RMS contrast and those normalised for apparent, perceived contrast. In each contrast condition, eight comparisons compared response times between upright fear and counterpart expressions (4) and again for control versions of faces (4). All comparisons were Šidák-corrected according to eight comparisons: α = 0.0063.

In summary, for LSF stimuli, fearful faces were not detected more quickly than any other expression, in any conditions.

### Response times for MSF faces

A repeated measures ANOVA showed significant effects of facial expression and orientation, and also an effect of contrast [*F*(4, 64) = 15.52, *p* < 0.001, η_p_^2^ 0.49; *F*(1, 16) = 6.28, *p* 0.02, η_p_^2^ 0.28; *F*(1, 16) = 49.81, *p* < 0.001, η_p_^2^ 0.75, respectively]. Overall, faces normalised for RMS contrast were more visible compared to those normalised for apparent contrast. Normal, upright faces were also more visible than the control versions. Contrast-expression, expression-orientation, and contrast-orientation interactions were all significant [*F*(4, 64) = 2.74, *p* = 0.03, η_p_^2^ 0.15; *F*(4, 64) = 3.70, *p* = 0.01, η_p_^2^ 0.18; *F*(1, 16) = 7.44, *p* = 0.01, η_p_^2^ 0.31, respectively]. No significant 3-way interaction was observed [*F*(4, 64) = 0.75, *p* = 0.56, η_p_^2^ 0.04].

Eight Šidák-corrected tests (α = 0.0063) compared RTs for fear to each other expression, both for upright and control faces. When normalised for RMS contrast, upright fear expressions are more visible than angry faces (*p* = 0.005), but this effect was not preserved for control versions of faces. When normalised for apparent contrast, there are no differences in RTs between upright fear and other facial expressions, but notably, RTs for control fear expressions were slower compared to happy faces. All comparisons are summarised in Table 3, and illustrated in Fig. 2c,g.

**Table 3:** Visibility differences between midrange frequency expressions normalised for contrast.

	*t*	*df*	*CI*	*p*
**Expression comparisons (RMS)**				
Fear–neutral	− 0.003	16	− 198.17, 197.68	0.99
Fear–anger	− 3.29	16	− 1259.86, − 273.95	0.005
Fear–happy	0.93	16	− 94.84, 244.35	0.36
Fear–disgust	− 0.14	16	− 307.50, 268.65	0.88
**Control comparisons (RMS)**				
Fear–neutral	2.57	16	30.11, 308.49	0.02
Fear–anger	− 2.21	16	− 567.99, − 12.89	0.04
Fear–happy	2.12	16	.52, 596.90	0.05
Fear–disgust	0.33	16	− 162.68, 222.97	0.74
**Expression comparisons (apparent)**				
Fear–neutral	0.48	16	− 277.19, 442.14	0.63
Fear–anger	− 2.79	16	− 796.04, − 109.47	0.01
Fear–happy	2.90	16	107.34, 690.07	0.01
Fear–disgust	− 0.07	16	− 410.74, 383.78	0.94
**Control comparisons (apparent)**				
Fear–neutral	2.96	16	157.16, 943.08	0.01
Fear–anger	0.79	16	− 243.47, 536.11	0.43
Fear–happy	4.61	16	476.03, 1284.62	< 0.001
Fear–disgust	2.68	16	101.94, 872.93	0.01

Pairwise comparisons conducted separately for faces normalised for RMS contrast and those normalised for apparent, perceived contrast. In each contrast condition, eight comparisons compared response times between upright fear and counterpart expressions (4) and again for control versions of faces (4). All comparisons were Šidák-corrected according to eight comparisons: α = 0.0063.

In summary, for MSF stimuli, n fearful faces were detected more quickly than angry faces, but only when matched for RMS contrast. When matched for apparent contrast, response times are slower for detecting fear than happy control faces.

### Response times for HSF faces

A repeated measures ANOVA showed significant effects of facial expression and contrast, but no significant effect of orientation [*F*(4, 64) = 22.92, *p* < 0.001, η_p_^2^ 0.58; *F*(1, 16) = 36.07, *p* < 0.001, η_p_^2^ 0.69; *F*(1, 16) = 1.28, *p* 0.27, η_p_^2^ 0.07, respectively]. Significant contrast-expression, expression-orientation, contrast-orientation, and 3-way interactions were observed [*F*(4, 64) = 2.93, *p* 0.02 η_p_^2^ 0.15; *F*(4, 64) = 3.91, *p* 0.007, η_p_^2^ 0.19; *F*(1, 16) = 48.12, *p* < 0.001, η_p_^2^ 0.75; *F*(4, 64) = 13.94, *p* < 0.001, η_p_^2^ 0.46, respectively].

Eight Šidák-corrected tests (α = 0.0063) compared RTs for fear to each other expression, both for normal upright and control faces. When normalised for RMS contrast, RTs for upright fear expressions were faster compared to both angry and happy faces (both *p* < 0.001). Only the effect between fear and anger remained true for control faces (*p* ≤ 0.001). When normalised for apparent contrast, upright fear expressions were detected faster compared to angry faces (*p* = 0.0060), but this effect diminished for control faces. Notably, control fear expressions were detected more slowly compared to neutral controls (*p* 0.001). All comparisons are summarised in Table 4, and illustrated in Fig. 2d,h.

**Table 4:** Visibility differences between high frequency expressions normalised for contrast.

	*t*	*df*	*CI*	*p*
**Expression comparisons (RMS)**				
Fear–neutral	2.22	16	11.65, 480.13	0.04
Fear–anger	− 7.71	16	− 725.77, − 412.94	< 0.001
Fear–happy	− 5.40	16	− 766.13, − 334.60	< 0.001
Fear–disgust	− 1.95	16	− 397.50, 16.13	0.06
**Control comparisons (RMS)**				
Fear–neutral	− 0.12	16	− 207.67, 184.51	0.90
Fear–anger	− 4.66	16	− 520.24, − 194.94	< 0.001
Fear–happy	− 0.33	16	− 251.02, 183.38	0.74
Fear–disgust	− 0.81	16	− 116.49, 51.66	0.42
**Expression comparisons (apparent)**				
Fear–neutral	0.28	16	− 249.76, 326.35	0.78
Fear–anger	− 3.13	16	− 720.03, − 138.66	0.0060
Fear–happy	− 0.74	16	− 432.89, 207.52	0.46
Fear–disgust	− 0.22	16	− 283.04, 228.26	0.82
**Control comparisons (apparent)**				
Fear–neutral	3.84	16	199.61, 690.45	0.001
Fear–anger	− 2.53	16	− 568.04, − 50.45	0.02
Fear–happy	− 1.25	16	− 455.39, 117.53	0.22
Fear–disgust	− 2.94	16	− 997.56, − 163.21	0.01

Pairwise comparisons conducted separately for faces normalised for RMS contrast and those normalised for apparent, perceived contrast. In each contrast condition, eight comparisons compared response times between upright fear and counterpart expressions (4) and again for control versions of faces (4). All comparisons were Šidák-corrected according to eight comparisons: α = 0.0063.

In summary, for HSF stimuli, fearful faces were detected more quickly than the original angry and happy faces, and angry faces only for the control facial stimuli. When matched for apparent contrast, they were only perceived more quickly than normal, angry faces.

## Discussion

The objective of the present study was to perform a replication and extension of the experimental design employed by Stein et al.^25^. This extension compared fearful faces to a broader range of spatially filtered facial expressions, included midrange, bandpass stimuli as well as lowpass and highpass images, and repeated the experiments for stimuli matched both for their physical RMS contrast, and for their apparent, perceived contrast.

Overall, our findings were broadly consistent with those of Stein et al.^25^ in that a bias for detecting fear expressions was found in high frequency conditions. Notably, Stein et al.^25^ study only compared spatially filtered fear and neutral expressions. Though we did not observe a significant difference between upright fear and neutral expressions at any frequency condition, at high spatial frequencies fear expressions *were* detected faster compared to both happy and angry expressions, and these effects were most pronounced when stimuli were normalised for RMS contrast. In this sense, our findings both support and extend those of Stein et al.^25^. To our knowledge, the present study and that of Stein et al.^25^ are the only ones to explore biases for spatially filtered expressions using b. CFS. Perceptual biases for fearful expressions are often found to rely on low frequency tuning, and so we propose that the effects observed in the present study, including those of Stein et al.^25^, are a facet of expression perception specific to the b. CFS paradigm. At broadband and low frequency conditions, we found rather limited evidence to suggest that fearful faces are perceived more rapidly than other expressions. Many studies evidence an initial fear bias for intact broadband stimuli^8,16–18,25,44^, though in the present broadband condition, detection advantages for fearful expressions were only found compared to anger, but not neutral, happiness, or disgust. Notably, this may be due to the number of expressions included in the present study, including stringent effects incurred from statistically-corrected comparisons for both upright and control faces. For more information regarding the statistical power of our study, please see Supplementary Tables 1 and 2.

We found that evidence for the threat bias was much diminished when stimuli were matched for apparent contrast, than when matched for RMS contrast. Normalisation for luminance and contrast is a routine procedure in studies of biases in the perception of facial expressions, since all other things being equal, brighter, higher contrast stimuli will be easier to see. Normalisation is therefore performed on the assumption that any such low-level differences between stimuli would artefactually influences the results. However, analyses of photographs have found naturally occurring differences in contrast across emotional expressions^36,37^. If the threat bias were to provide a behavioural advantage in everyday life, then it should be evident without prior contrast normalisation, particularly given these reliable differences between expressions. This analysis also showed a difference in the Fourier amplitude slope, with fearful faces having a steeper slope than other expressions^37^. This means that fearful expressions have relatively low contrast at high spatial frequencies. This means that, when normalising for RMS contrast in broadband stimuli, the amplitude of *all* frequency bands will be increased. This is important because the root cause of the reduced contrast in fearful faces is found primarily in a frequency band that contributes little to apparent contrast, but the normalisation will increase the contrast at low to midrange frequencies, known to be important in both apparent contrast^35^ and in the threat bias^8^.

Our finding that the threat bias is most evident at high spatial frequencies is consistent with the findings from Stein et al.^25^, but is at odds with those derived from non-b. CFS studies that show a low frequency role for fear biases. Across studies using different behavioural tasks, there is a wide variation in the spatial scale of information driving the effects, and thus the corresponding neural mechanisms that have been implicated. The threat bias found in b. CFS appears to be driven by high spatial frequencies. This information is processed by the parvocelluar layers of the LGN, and Stein et al.^25^ outline how this information would then be processed by cortical mechanism. This would most likely be via the distributed cortical network of brain areas involved in the processing of faces, in the higher-level, ventral regions of the visual cortex^45,46^. Conversely, results from other tasks including saccadic latency show that orientation towards images of faces^8^, and also the orientation of spatial attention and spatial sensitivity^13^, have been associated with the subcortical processing of low spatial frequency in areas including the amygdala^21^. Finally, it has been suggested that the preferential processing of fearful faces reflects the fact their spectral content is especially well matched with the contrast sensitivity function of the human visual system^18^. This matching relies on the fact that fearful faces have increased energy at midrange spatial frequencies, once matched for RMS contrast. The contrast sensitivity function is determined by properties of visual processing at a range of levels, including the centre-surround properties of cells retinal geniculate cells^47^, and the sampling of spatial frequency in the primary visual cortex^48^. The visual processing associated with increased salience of fearful faces thus runs through from the retina, subcortical areas, primary visual cortex and higher visual areas in the ventral stream, and visual information spanning low, midrange and high spatial frequencies. Rather than reflecting a single stage of visual processing, responses to emotional expressions occur at multiple levels of processing, involving a complex network of forward, lateral and backwards connections across levels^49^. There appears to be no single, well-defined adaptation that might be expected to provide a broad behavioural advantage in our responses to fearful faces.

Together, the present findings highlight the combined effects of spatial frequency and contrast on face visibility under b. CFS. They show, along with other recent findings, that routine experimental procedures such as contrast normalisation can have facilitatory effects on stimulus salience^37,50^. Moreover, they contribute to current and developing discussions regarding the mechanisms of b. CFS, its reliability as a measure of conscious visual processing^23,24,26–29,38,51^, in showing that stimulus visibility under b. CFS also varies according to the contrast content of face stimuli, and the implications this has for our understanding of perceptual biases for emotional expressions.

## Supplementary information

Supplementary Information.
