# Supplementary material for: Suppression durations for facial expressions under breaking continuous flash suppression: effects of faces’ low-level image properties
Source: Sci Rep. 2020 Oct 15;10:17427. doi: 10.1038/s41598-020-74369-2 (PMC7567108; doi:10.1038/s41598-020-74369-2)
Supplement: Supplementary file 1 — Supplementary Information. [file 41598_2020_74369_MOESM1_ESM.pdf]

Supplementary Information

Suppression durations for facial expressions under breaking continuous flash suppression: effects of faces' low-level image properties.

Abigail L. M. Webb & Paul B. Hibbard

Analyses of power

We decided that an analysis of power was not possible because, to our knowledge, only one other directly comparable study currently exists (Stein et al., 2014). There was therefore not possible to retroactively conduct a power analysis, there was insufficient pre-existing data required.

We also decided against conducting a retrospective analysis of power, on the basis that it would not be appropriate for two reasons: (1) such a power analysis would be retrospective, and (2) our findings were broadly consistent with those of Stein et al. (2014); the only study most comparable to our own i.e. using b. CFS to explore face perception using spatially filtered faces. We therefore do not feel that there is substantial motivation for conducting a post-hoc power analysis. Instead, we have taken an alternative approach to addressing the power of the study, comparing the sample and effect sizes of our study with those of other comparable studies. Below, Supplementary Table 1 displays details of pre-existing studies most comparable to our own in terms of design and analysis i.e. studies of facial expression detection (including fear expressions) under b. CFS, where the analysis includes an assessment of the main effect of expression. It includes sample sizes, details regarding the main effect of expression, statistical significance and effect sizes (partial eta-squared). As a reminder: our broadband face study included 29 observers, and showed a significant effect of expression: $F(4, 112)= 22.59$, $p <.001$, η_{p2} .44. For our low, midrange, and high frequency conditions, main effects of expressions were: $F(4, 64)= 3.85$, p .007, η_{p2} .19; $F(4, 64)= 15.52$, $p <.001$, η_{p2} .49; $F(4, 64)= 22.92$, $p <.001$, η_{p2} .58, respectively).

The information summarised in Supplementary Table 1 shows that with the exception of one study (Gray et al., 2013), our study of *broadband* facial stimuli had a sample size above that associated of the other 4 most comparable studies, but our obtained effect size (η_{p2} .44) was lower than that of all but one study (Hedger, Adams & Garner, 2015). Notably, Hedger and colleagues (2015) state that their own sample size (n=22) was sufficient to detect a large effect size for performance differences between broadband expressions under b. CFS, based on expression comparisons under b. CFS by Yang and colleagues (2007). A priori, given the similarity between our experimental design and that of Yang et al. (2007) and Hedger et al. (2015), our study power should have been sufficient to detect such an effect. Moreover, our frequency filtered conditions (low, midrange, and high frequency faces) have a larger number of participants than Stein et al. (2014), though our effect sizes are smaller (see Supplementary Table 1 for details). Moreover, our results are broadly consistent in terms of the direction of effects: for our broadband facial stimuli, we show that fear is advantaged in overcoming b. CFS mask suppression compared to angry faces when statistical comparisons were corrected using Sidak-corrections. The non-significant differences, however, also followed similar patterns, where fear generally appeared to be detected faster compared to other expressions. It is possible that these did not reach statistical significance due to the number of corrected comparisons we conducted.

In terms of frequency filtered faces, although other psychophysical studies have shown a fear advantage that is emphasised when faces are low frequency filtered, we did not find this effect. Instead we found the opposite effect, consistent with Stein et al., (2014). To our knowledge, Stein et al., (2014) is the only other study to use b. CFS to measure fear detection advantages using spatial filtering. It may therefore be that spatial frequency tuning for face perception under b. CFS relies on spatial frequency information in a way that is different under other non-b. CFS conditions.

Finally, Supplementary Table 2 lists non-b. CFS studies that explore fear bias effects using spatially filtered facial expressions. Again the include effect sizes where possible, for those cases where significant differences were reported. Notably, because data are from non-b. CFS studies (saccadic latency, gender discrimination, basic localization, and spatial attentional priming studies) they do not serve as useful comparisons to our own study. Stein et al., (2014) is directly comparable in terms of design, and given that the authors' report large effect sizes using a smaller sample than our own. We therefore conclude that our own study was sufficiently powered. Note also that Supplementary Table 2 includes studies of behavioural data only. Neuroimaging evidence is not included here, where much of the low frequency threat bias support come from.

Supplementary Table 1. Details of effect and sample size from similar studies concerned with facial expression detection under b. CFS conditions. In instances where effect sizes are not provided by authors, we have calculated these based on output F-statistics (Lakens, 2013), as partial eta-squared as this is the effect size adopted in our own study. Note: details of Jiang et al. (2009) are omitted, on the basis that it the study does not provide behavioural response data (ERP data only).

**Authors (year)**	**Study no.**	**Sample (n)**	**Expressions**	**Analysis info.**	**Main effects of expression**	**Notes**
Stein et al. (2014)	1a	12	Fear, neutral	Repeated measures ANOVA	- At BSF fear breaks suppression faster than neutral, ($F(1, 11) = 25.03$, $p<.001$, η_{p^2} **.69**.<br>- At HSF fear breaks suppression faster than neutral, ($F(1, 11)= 39.20$, $p<.001$, η_{p^2} **.78**.*<br>- At LSF: no significant effect<br><br>* **effect sizes** calculated as partial eta sq.	We only compare data from experiment 1a, as this was a straight-forward filtered face detection task under b. CFS, and thus is the one that best matches our own design. Studies 1b-c and 2 were concerned with different methods for masking and/or use hybrid facial stimuli.
Gray et al. (2013)	3	41	Fear, neutral, angry, happy	" (assumed)	- At BSF expression effect, where fear detected fastest ($F(3, 108)=53.36$, $p<.001$, η_{p^2} **.61**	Study only measures intact broadband faces

Yang, Zald & Blake (2007)	1	12	Fear, neutral, happy	Repeated measures ANOVA	-	At BSF expression effect, fear breaks suppression fastest (F(1.6, 16.64)=13.67, P<.001, **η_{p}^{2} .56*** <br>* **effect sizes** calculated as partial eta sq.	Study only measures intact broadband faces
Yang, Zald & Blake (2007)	2	14	Fear, neutral, happy	Repeated measures ANOVA	-	At BSF expression effect, fear detected fastest, **η_{p}^{2} .74.*** Followed by comparisons showing fear as sig fastest. <br>* **effect sizes** calculated as partial eta sq.	Study only measures intact broadband faces
Hedger, Adams & Garner, 2015	2	22	Fear, neutral, happy, angry	" (assumed)	-	At BSF expression effect, fear detected fastest (F(3, 63)= 5.33, *p*.002, **η_{p}^{2} .20*)** <br>* **effect sizes** calculated as partial eta sq.	Study only measures intact broadband faces. Authors note that "sample size provides in excess of 95% power to detect a large effect size, Cohen's *d* 1.15 (the magnitude of difference in detection between fearful vs. neutral faces from a similar CFS paradigm; Yang et al., 2007)."

Supplementary Table 2. Details of effect and sample size from similar studies (non-b. CFS) concerned with perceptual biases for responding to spatially filtered fear expressions. In instances where effect sizes are not provided by authors, we have calculated these based on output F-statistics (Lakens, 2013), as partial eta-squared as this is the effect size adopted

Authors (year)	Study no.	Sample (n)	Expressions	Analysis info.	Main effects of expression		Paradigm/Notes
in our own study.							
Stein et al. (2014)	3	16	Fearful only	Repeated measures ANOVA (assumed)	-	Overall expression effect, where high frequency fear bybrids more accurately detected than low frequency counterparts (*F*(1, 15) = 7.61, *p* = .015, **η_{p}^{2} .33*)**<br><br>*** effect sizes** calculated as partial eta sq.	**Hybrid face detection under forwards/backwards masking conditions**. No response time data provided. No neutral face comparison.
Pourtois et al. (2005)	1	13	Fearful, neutral	Repeated measures ANOVA	-	Accuracy and response times best for unfiltered faces. No significant performance difference between low and high hybrid faces.	**Gender discrimination task with face hybrids.**
Bannerman et al. (2012)	1	28	Fearful, neutral, happy	Separate one-way ANOVAs/frequency con.	-	At BSF expression effect, SRTs faster for	**Saccadic response times (SRTs) for localizing filtered faces.**

					fear than neutral but not happy faces *F*(2, 54) 6.68, *p* .01, **ηp2 .20.**<br>- At LSF expression effect, SRTs faster for fear than neutral and happy *F*(2, 54) 10.84, *p* .001, **ηp2 .29**<br>- At HSF, no significant expression effect.	
Vuilleumier et al., (2003)	1	13	Fearful, neutral	Repeated measures ANOVA (assumed)	- No significant expression effects for accuracy or response time.	**Gender discrimination task for filtered facial expressions.**
Holmes, Green & Vuilleumier (2005)	1	28	Fearful, neutral	Mixed ANOVA	- At LSF, RTs to fearful at same location as probe were faster than neutral counterparts<br>- At HSF, no significant expression effect<br>- Reported as frequency by	**Attentional cueing with spatially filtered faces.**

					expression location interaction, $F(1, 26) = 4.84$, $p < .05$, η_{p}^{2} .16	
Holmes, Green & Vuilleumier (2005)	4	12	Fearful, neutral	“	- No significant differences or interactions for frequency and/or expression	**Localising fear expressions in spatially filtered face pairs**
Holmes, Green & Vuilleumier (2005)	5	31	Fearful, neutral	“	- Reports non-significant interaction between frequency and expression. But also reports:<br>- At LSF, RTs are faster on trials where lSF fear rather than neutral precedes target, $t(15) = 2.2$, $p < .05$<br>- At HSF, no effect of expression	**Attentional cueing with spatially filtered faces**

1. Bannerman, R. L., Hibbard, P. B., Chalmers, K., & Sahraie, A. (2012). Saccadic latency is modulated by emotional content of spatially filtered face stimuli. *Emotion*, *12*(6), 1384.

2. Daniel Lakens 2013, *The first rule of not understanding effect sizes is you don't talk about not understanding effect sizes*, Daniel Lakens, viewed 27th May 2020, < https://sites.google.com/site/lakens2/blog/thefirstruleofnotunderstandingeffectsizesisyoudon%E2%80%99ttalkaboutnotunderstandingeffectsizes>
3. Gray, K. L., Adams, W. J., Hedger, N., Newton, K. E., & Garner, M. (2013). Faces and awareness: low-level, not emotional factors determine perceptual dominance. *Emotion*, *13*(3), 537.
4. Hedger, N., Adams, W. J., & Garner, M. (2015). Fearful faces have a sensory advantage in the competition for awareness. *Journal of Experimental Psychology: Human Perception and Performance*, *41*(6), 1748.
5. Holmes, A., Green, S., & Vuilleumier, P. (2005). The involvement of distinct visual channels in rapid attention towards fearful facial expressions. *Cognition & Emotion*, *19*(6), 899-922.
6. Pourtois, G., Dan, E. S., Grandjean, D., Sander, D., & Vuilleumier, P. (2005). Enhanced extrastriate visual response to bandpass spatial frequency filtered fearful faces: Time course and topographic evoked-potentials mapping. *Human brain mapping*, *26*(1), 65-79.
7. Stein, T., Seymour, K., Hebart, M. N., & Sterzer, P. (2014). Rapid fear detection relies on high spatial frequencies. *Psychological science*,*25*(2), 566-574.
8. Vuilleumier, P., Armony, J. L., Driver, J., & Dolan, R. J. (2003). Distinct spatial frequency sensitivities for processing faces and emotional expressions. *Nature neuroscience*, *6*(6), 624-631.
9. Webb, A., & Hibbard, P. (2020, May 26). Contrast normalisation masks natural expression-related differences and artificially enhances the perceived salience of fear expressions. Retrieved from osf.io/hkrd9
10. Yang, E., Zald, D. H., & Blake, R. (2007). Fearful expressions gain preferential access to awareness during continuous flash suppression. *Emotion*, *7*(4), 882.
